# Born in Captivity: The Experiences of Puerto Rican Birth Workers and Their Clients in Quarantine

**DOI:** 10.3389/fsoc.2021.613831

**Published:** 2021-03-12

**Authors:** Emaline Reyes

**Affiliations:** Department of Anthropology, Temple University, Philadelphia, PA, United States

**Keywords:** COVID-19, Home birth, midwifery model, reproductive justice, obstetric violence

## Abstract

In this article, I seek to understand how the COVID-19 pandemic has impacted childbirth in Puerto Rico, an island that was already in recovery following the occurrence of two devastating hurricanes in the fall of 2017 and a major earthquake in the winter of 2020. Thus, I argue that it is important to discuss not only how individual disasters impact birth, but also how their compounding effects do so. In order to address these research questions, I conducted remote interviews with Puerto Rican birth workers and researchers. During times of crisis, this pandemic included, home and midwife-attended births have become increasingly more popular. However, Puerto Rican midwives and doulas currently have less institutional support than ever. In a time of quarantine when home births are rising, we need to consider whether society is designed to facilitate these models of care. In Puerto Rico, pre-pandemic, there was a less than 1% home birth rate and there still is a lack of legal recognition and protections for homebirth midwives. As this article demonstrates, an acknowledgment of the near-invisible labors of these birth workers is needed, in addition to supplies, support, and protections for them—and not just in times of “crisis.”

## Introduction: Disasters and Deliveries

From Ebola to Zika, a primary concern in recent epidemics has been how infectious disease impacts pregnant women and infants. The COVID-19 crisis is no different, with headlines across the nation reporting on women who have decided to labor in their homes (Freytas-Tamura, 2020) or on doulas who were barred from entering hospitals (Meyerson, 2020)—in addition to the more biologically-focused questions of how COVID impacts the pregnant body and infant development. Puerto Rico, a US territory with a legacy of colonial control and exploitation, is in a particularly vulnerable position when it comes to COVID and maternal wellbeing, as its citizens have historically struggled with reproductive justice and access to adequate healthcare (Briggs, 2002; Lopez, 2008; Córdova, 2017). Most recently, before COVID, Puerto Rico was in the process of recovering from the 2017 Hurricanes Irma and Maria and the 2020 earthquakes. These left the population in a precarious position in regard to their economy and infrastructure and have certainly complicated their COVID responses (from what I have observed). What we see in Puerto Rico are incomplete recovery and the compounding effects of multiple and ongoing disasters. I have found that the impacts of COVID on childbirth in Puerto Rico are similar to the impacts seen in those prior disasters, and that the responses to these events have been strikingly similar as well. Furthermore, I argue that these patterns seen in disaster response and experience in Puerto Rico reflect the patterns observed in other disasters in other countries (Wick and Hassan, 2012; Ivry et al., 2019; Saulnier et al., 2020; Davis, 2021), now including COVID. In what follows, I will discuss how these disasters, and in particular COVID, have been lived, experienced, and managed—paying special attention to how COVID-19 has impacted childbirth in Puerto Rico, as well as maternal care systems and the labors of local birth workers who are often on the frontlines of disaster care.

## Background

When Hurricanes Irma and Maria hit Puerto Rico in 2017, many laboring women were unable to get in touch with their primary providers or to get to the hospital (Stein, 2017). During this time, midwives were on the frontlines, leading disaster response and relief both in maternity and community care (Dieppa, 2018), as midwives in other countries have also frequently done^1^. Midwives and home births in Puerto Rico were being covered by the mainstream media in ways previously unseen, creating more awareness of the work that homebirth midwives do and the diverse birth options available (to some) (Liautaud et al., 2017). Independent midwives were assisting in more home deliveries than ever before. There are only about two dozen practicing independent midwives in Puerto Rico, all of whom are certified professional midwives (CPMs), who are not allowed to practice in hospitals, and the pre-COVID homebirth rate in Puerto Rico was <1%; it remains to be seen how much that rate has risen during the pandemic^2^. Less than 2.5 years after Hurricanes Irma and Maria, when extreme earthquakes rocked the southern region of the island, these independent midwives were once again first responders. Though they are adamant that no births occurred in the “tent cities” (due to safety concerns), they did provide prenatal and postnatal care for the women of the community—many of whom were still having difficulty recovering from the earthquakes^3^, both emotionally and physically. During COVID, we once again observe an increased reliance on midwives and a shift of births from the hospital to the home, due in large part to fears of infection, similar to the fear of leaving the home witnessed at the height of the Zika epidemic (Rodriguez, 2017). But we also must acknowledge the complications in this shift, for both midwives and their clients, as I will later discuss.

## Methods

In order to understand how COVID impacted birth in Puerto Rico, and how this differed from or was similar to previous disasters, I conducted remote interviews with 11 Puerto Rican women working in the fields of reproductive health and justice, including: 5 midwives, a doula, a clinical psychologist working in a Neonatal Intensive Care Unit (NICU), a social worker focused on combating gender violence, a child birth photo journalist, a midwifery student, and a fellow researcher. As midwives, doulas, and their allies are often found on the frontlines of disasters, working directly with laboring people, they are most knowledgeable about how these adverse events impact birth. I conducted these interviews via Zoom and on the phone, primarily in English (a second language for the birth workers) with some Spanish spoken intermittently. Individual interviews were conducted multiple times to track how the pandemic and responses to it changed over the April to August 2020 timespan. During this time, I closely monitored the media on birth experiences in quarantine and continued to review the literature on maternal services in times of disaster.

This project was granted exemption status from the Temple University Institutional Review Board because it consists entirely of interviews that are more in the tradition of journalism or oral history (and thus do are not subject to IRB oversight), and because there are adequate provisions in place to protect the privacy of any respondents who wish to remain anonymous. Verbal consent was obtained.

## Findings: Birth Location, Power Dynamics, Social Realities, and Past, Present and Future Issues Around COVID-19, Midwives, and Birth

Interview results were mixed, with some stories of difficulty and despair and some messages of hope and resilience. I will describe and analyze these findings in terms of four emergent themes: birth location; vulnerability and isolation; advantages and disadvantages of telehealth; and midwives’ reaction to obstetrician’s increased domain protection. The information presented below comes directly from the accounts of my interlocutors (unless otherwise cited).

### Locating Birth

Regarding birth location, my midwife interlocutors informed me that home births have become more popular and by extension, their services have been in high demand. However, they note that these services are still not accessible to everyone. This is especially true for those of a lower socioeconomic status—whether they want to or not, often they have no choice but to deliver at no cost in the public hospitals, where COVID cases are worst. The exceptions are those who are able to access free or reduced-cost midwifery and doula services, which some of my interlocutors offer. This issue of access is due to both the lack of insurance coverage of midwives and home birth and to the lack of stable housing and necessary resources such as clean water, clean surfaces, and supplies. Many people lost their homes during the hurricanes and the earthquakes; home birth is impossible for those who are home-*less*, and hospital and clinic births are the default. Even for those who have homes, blue tarp “roofs” are not uncommon following Maria, and the additional economic constraints put in place by COVID have made it difficult to access necessary supplies and safe, secure, sanitary spaces.

For those who *are* able to give birth within the home, home births and midwifery services have been significantly altered by the virus, as my interlocutors have explained. Midwives must wear gowns, face masks and face shields, and of course gloves. If they are visiting homes for prenatal or postnatal check-ups in person, they must practice social distancing, wash their hands persistently, and keep hand sanitizer with them at all times. Some are simply using telehealth for these appointments (leading up to and following delivery). During labor and birth, all midwife care must obviously be administered in person, cautiously. Similar to the in-person appointments, social distancing guidelines are followed, and protective gear is worn. Midwives keep their distance as much as they can, relying heavily on the family to offer physical support to the laboring woman. They have to be close when the baby comes, so that they can catch and attend to it, but some say that they have heard stories of women being taught to catch their own babies while under the supervision of trained midwives. The company of healthy family members and doulas is encouraged, but smaller group sizes are preferred. This differs drastically from pre-COVID home births in which family, friends, and neighbors alike were all encouraged to attend, and midwives provided hands-on care throughout.

Additionally, for the protection of midwives, their clients, and the community, the screening process is much more intense—including inquiring about COVID symptoms and contact with infected individuals. Many midwives had to wait for their own test results before they could serve their clients. I was not told whether or not these midwives required that their clients get tested, but one midwife informed me that the midwives have great difficulties in accessing rapid COVID tests. Other articles in this Special Issue echo the accounts of my interlocutors.

While home birth may be challenging amid COVID, for those who give birth within the hospital, labor can be even more challenging. My interlocutors have reported that, unfortunately, during this time, cases of obstetric violence have increased. The isolation of birthing women has most likely contributed to this problem. Doulas are being banned in most hospitals on the island (with the exception of one hospital, identified by a doula I spoke with) along with other support persons. The lack of company, advocacy, and thus provider accountability has left many women vulnerable to the abusive practices of hospital personnel. Yet part of the poor treatment and care that women are receiving in hospitals is due to negligence rather than direct abuse. Hospitals are overburdened by COVID and were already dealing with a shortage of personnel as a result of the mass exodus that followed Hurricane Maria. One midwife told me about a client of hers who was transferred to the hospital, only to have her baby die there:

One of my clients called me in May and said she hadn’t felt the baby move. I went to check up on her and could hear movement but could also tell that something was wrong. So I took my client to the hospital and called and told them about what was going on and how this woman needed a nurse to check her vitals and physically examine her...the thing is they (the hospitals) aren’t hiring nurses and other personnel like they should be. So, I spoke with a nurse and said, “I’m sending my client and you need to do these things” and she (my client) showed up at the hospital and they didn’t do anything.

Guess what? There was one nurse on that floor. In the end the baby died in the hospital, she had a stillbirth. They (the hospitals) need more people, things would have been different if there was a nurse who could have attended to my client. It probably would have been a C-section, but that baby would be alive today. There are less and less people in the hospitals. This has been such an issue and continues to be one. And the people who are there are giving worse care too. They are overextended because so many left—the mass exodus after Maria. You can feel that.

While this midwife wished to remain anonymous in regard to this specific account (for fear of retaliation from the clinical community--a conflict resulting from the power struggle between midwives and clinicians that will be discussed below), she was determined that I share it, noting how crucial it is for people to hear about these preventable tragedies. I was told that, shockingly, some maternal deaths may have been attributed to COVID with insufficient evidence. Midwives fear that the virus is being used to cover up malpractice and justify any maternal deaths for which the hospital does not want to be held liable. COVID has also given practitioners justifications for many unnecessary and excessive practices; when negligence is not the issue, increased intervention is. Most notably, the performance of more inductions and cesareans is being reported, with the justification that these move women more quickly through the system, thereby limiting potential viral exposure (Davis-Floyd, 2021; Davis-Floyd et al., 2020). Mothers and infants are often separated following cesarean birth, and even when delivered vaginally in hospitals, many mothers and infants are being separated post-birth due to fear of COVID transmission, though *in the absence of a positive test result or evidence that demonstrates mother-infant viral transfer.* Immediate skin-to-skin contact is thus often disallowed, making breastfeeding initiation difficult, both of which can seriously impact maternal-infant health and relationships, as well as postpartum recovery, and can force use of expensive formula often unavailable to the poor, or can result in cheap powdered formula being mixed with contaminated water—a potentially deadly situation for infants.

When I discussed this issue with a clinical psychologist, she was adamant that the harm of initial separation is not irreparable—you can still bond and breastfeed once you are finally reunited with your infant. She reminded me that this resilience perspective is crucial for survival, something she learned working in the NICU:

I do believe in immediate breastfeeding initiation and skin-to-skin contact and bonding, but I think that we need to be very careful to communicate to mothers that they can still connect and still breastfeed even after separation. They do not “miss” that one opportunity, they can make the best of the situation from there...I guess I have this perspective from working in the NICU, because (compared to these most recent COVID separations) we *always* have to separate the mothers and infants and yet we still try to encourage these practices (breastfeeding, skin-to-skin) in the long run. Even if a baby has been in the NICU for 7 months they can develop a close relationship with their parents and can still be successful in breastfeeding after they finally get to go home...this is difficult for people to comprehend if they haven’t seen it. But we’ve seen it, and we know.

While birth location can certainly impact experiences and outcomes, it is important to remember that these beneficial practices can be utilized and encouraged at various stages and in many places.

### Cultural and “Covidian” Concerns for Homebirth Midwives and Their Clients: Vulnerability and Isolation

Puerto Rican midwives have worked tirelessly to gain respect and recognition. In addition to navigating a pandemic, they must also dispel myths that midwives are “dirty,” unsanitary, uneducated, and ill-equipped.” They face stereotypes that date back centuries in Latin America and the Caribbean and are often associated with the race and ethnicity of the midwife, especially African ancestry and Indigeneity. Even as their work becomes more visible, local birth workers such as midwives and doulas are not recognized as healthcare professionals; therefore, they have not been given the same support (financial and political), supplies, and PPE as other healthcare workers. This has left them in a very vulnerable place, given that their services are in demand now more than ever. And it is also a dangerous time for them to be working and coming into contact with so many different people (their patients, their patients' families). Midwives worry about contracting COVID, both for themselves and for the safety of their own families. They talk about having to take everything off before entering the home, having to shower thoroughly and wash laundry multiple times, being cautious around their own children. Many worry that midwives will contract COVID at higher rates than any other essential workers. One midwife I spoke with, Tamara, wondered how many COVID cases we would see among midwives in the coming months:

Even if the Puerto Rican government doesn’t want to acknowledge that we’re health providers, we *are*. Midwives are acknowledged in other parts of the world, but they’re not *seen* here. Because we aren’t classified as healthcare providers, disaster funding goes to doctors but not to us. We are working for free and risking ourselves...We are having a lot of difficulty accessing COVID rapid tests. . .Because we don't have access, we are exposing ourselves going into the houses of these women who need our help. Other healthcare providers are given the tests and essential supplies but we have only gotten them through donations (we have had to buy supplies as well)...I think in September or October we could look back, gather our statistics, and say “our midwives got sick even when they used protection” because no one supported us. Right now, we’re dealing with something that affects us all and it’s pretty darn scary.

This constant stress and anxiety can take a serious toll on the mental health of community birth workers, as can the physical distance from their patients and the separation/disconnection that they feel due to protective measures such as masks and face guards. A number of midwives told me that one of the most difficult things about COVID has been not being able to embrace the baby after it is born. It saddens them that they cannot hold it, hug it, smell it, place a kiss on its forehead. There is sadness for both the mothers and the midwives in these home births.

COVID can be an incredibly lonely and isolating time, not only for midwives, but also for those who are about to or who have just given birth. One interlocutor told me that in Puerto Rico, pregnant women and new mothers are more than anyone else are self-isolating; they are worried for themselves, their babies, and their families. This isolation and constant worry can be detrimental to the mental health of both pregnant women and new mothers. What used to be a celebration is now a time of grave concern. They are lacking support in so many ways. They cannot have their older relatives (parents, grandparents) present for the birth; they must be careful of how many people they allow to attend the delivery or visit afterward, and they even have to be cautious around their own birth workers. One midwife, Gina, told me that one of her clients rejected postpartum care because she was so fearful of having anyone (other than herself and her immediate family) come into contact with her newborn baby:

Pregnant families are taking care of themselves the most. They are the ones quarantining in the house. I had a client who canceled her appointment with me 6 weeks away because she was worried about COVID. I had worked with her leading up to her birth, which ended up being a c-section, but even after a c-section 1 do the postpartum care. She declined these last visits because she wanted to protect the baby. She wasn’t letting anyone visit, even me as a midwife. But I respected her wishes and said “Okay. I hope you are both safe and healthy.”

In this general environment of fear and uncertainty, mothers are anxious about bringing children into this world and about allowing them to interact with others. This stress surely impacts the birth experience itself. Many mothers in Puerto Rico and elsewhere are dealing with postpartum depression, which is not uncommon following a trauma or disaster, but has been compounded by the crushing loneliness and despair of COVID. And quality of care can be seriously compromised—especially in the hospitals; some women are coming out of their pandemic hospital births more traumatized than ever due to the obstetric violence—and/or the neglect--they experience there.

### Telehealth: Advantages and Disadvantages

Over time, as the virus has been better understood and managed in some regions, families have begun to feel slightly less anxious. As government restrictions on the island have begun to relax, so too have citizens. Accordingly, some women may begin to feel less fearful, which could significantly reduce the stress that has been present in their deliveries. Most hospitals have started letting support persons into the delivery room (at the least a family member/partner), with the exception of one hospital (identified by my hospital doula contact) that continues to ban any support persons.

The telehealth journey has been particularly interesting, with arguments for both its advantages and disadvantages. For medical professionals, the implementation and use of telehealth could be seen as shifting power differentials. Patients are now in the position where they are the ones taking their own vitals, monitoring their own pregnancies, and guiding their own (virtual) appointments, leading up to and in preparation^4^ for birth. This is a level of patient agency and autonomy previously unseen in Puerto Rican maternity care, and in most of Western medicine as well. In this way, telehealth could be considered empowering for patients. One midwife, Yariana told me:

With telehealth and remote appointments there is a lot of emphasis on self-care and being aware of your health-it is empowering for women. Parents use to be taught how to take their blood pressure and things like that, but now they are learning about fetal heart monitoring and measuring their own belly. Telehealth, telemedicine teaches them (parents) those things. Being very hands on with health can change a lot in you and make you very conscious.

This account was echoed by the researcher I spoke with, and these initial interactions had me under the impression that telehealth could be a positive experience within the clinical context; however, some midwives worry that telehealth may actually be abused by medical professionals. They are concerned that doctors are exploiting this new system in order to “not do their job and still get paid,” and that now that doctors realize this is an option and something they could have been doing all along, they will continue to find reasons to do it more and more. This was the sentiment of one midwife I spoke with, Zulgeil:

I don’t like to use telehealth; I think it is pretty dangerous actually. I worry that it’s being abused by a lot of the doctors who are using it. Because they think, “oh, I don’t even have to touch someone, and I can still make money from my home?” It’s negligent really. I will do my initial interview through telehealth, but I will not recruit clients online, and then after that I will go in person and wear a mask and screen the clients and discuss ethics and protocols. But these doctors, they were doing 5-min appointments anyway, even before COVID! Now it’s like nothing. I mean I know some midwives are doing it, especially birth centers, and it’s a choice, but I’m just not comfortable with that. I’m following protocols in person, and anything I can do additionally online, I will. I just worry that now that telehealth is an option, it is just going to continue to be abused.

This is a legitimate concern that Zulgiel spoke to, and while telehealth is primarily used for prenatal and postnatal check-ups (with the exception of its use by doulas and other support persons in labor-a practice intended to circumvent restrictive hospital policies, or even to ensure proper social distancing in home births), limited attention in birth and lack of continuity of care can be real issues in the hospital, just as brief, condensed appointments are.

For midwives, telehealth has had little impact on the power differentials between practitioners and patients, as midwives already prided themselves on embracing a model of care that treated mothers as equals. Even before COVID, midwives were conscientious in supporting mothers to be active agents in their own pregnancies and births, constantly educating and involving them in decision-making. This client involvement is still there; however, some midwives argue that COVID, social distancing, and telehealth have impacted crucial patient-practitioner interactions in more negative ways. The midwifery model of care is based on humanistic and holistic care that relies heavily on physical, social, emotional, mental, and spiritual support (see Davis-Floyd 2018a for a full description of this model). Midwives argue that with telehealth and social distancing, this presence is missing, and, equally as important, so are their keen, trained eyes and informed touch. While the midwives I spoke with acknowledge the competence and intelligence of their patients, trusting them to take charge of their own care, they argue that this is their job, this is what they are “paid for”^5^, this is the kind of care they are supposed to offer (and what separates them from medicalized practitioners). They are adamant that they simply need to be there, to be able to offer hands-on support. The lack of their physical presence and ability to connect as human beings is detrimental both to these midwives and to their clients and could potentially negatively shape birth experiences.

### The Home/Hospital and Midwife/Obstetrician Divide: Obstetrician’s Increased Domain Protection and Midwives’ Responses

The pandemic has impacted the medical culture in Puerto Rico, emboldening doctors to “protect” their “domain,” which appears to be threatened by the growing preference for midwives. While some few humanistic obstetricians (one midwife approximated that their number was somewhere close to 4 out of 84) are being supportive of community birth workers during this time, especially doulas (whom they see as allies)^6^, overall, COVID has only served to further the divide between midwives and medical professionals. I have been told that this conflict existed before, but is exacerbated in times of crisis, including during COVID. From the very beginning of the pandemic, OBs have seen how preference for midwife-attended home births has increased and have responded by launching a campaign against home deliveries and the midwives performing them. In an extreme overreaction on an island with only 24 homebirth midwives and a pre-pandemic homebirth rate of 1%, medical professionals have taken to social media, recording videos through Facebook Live, and have gone on the news to argue that the absolute safest place to give birth is the hospital. They claim that this is especially true given the current pandemic, which they believe has made birth even *more* risky and pathological (more of a disease, more worthy of being medicalized). The midwifery student whom I spoke with explained to me that:

Parents are being guilted if they choose home birth. Doctors tell them that this is dangerous, negligent, abusive. They say it is the start of bad parenting and a bad childhood...many of these arguments are not evidence-based, they are fear-based. This is not, and should not be, a moral issue. This pressure was already there, but COVID is just a way in which they (the doctors) are reinforcing their message, which they already felt justified in promoting. Some people are emboldened during the time of the virus though

Some midwives worry that the precautions taken during COVID are leading the way to midwifery becoming “too clinical.”^7^ What makes homebirth midwifery so special and unique, they argue, is that it is based on humanity and spirituality in a way that sets it apart from the clinical, technocratic model (Gaskin, 2002; Davis-Floyd, 2018a; Davis-Floyd and Davis, 2018). Midwives distinguish between those who choose home birth because they truly want “natural” births and those who are just afraid of hospital contagion; they are unsure whether the increased preference for home birth and doula-attended births^8^ that they are currently seeing will persist, or whether this increase is a temporary result of the pandemic. Issues of accessibility also may determine whether or not higher rates of midwife-attended home births will continue over time, and many in Puerto Rico also want freestanding birth centers, yet so far none have emerged.

Most midwives want to be able to work alongside the local institutions, rather than against them, and they do want institutional recognition, but they also realize that such recognition may risk the values and standards they have set for themselves as community birth workers. Gaining the approval of the government will mean that they will essentially have to unionize, offer standardized care, all be on exactly the same page, and be governed by a set of laws/principles and protocols imposed by technocratic medicine^9^.

Additionally, it would be difficult for midwives to work with an institution that does not want to work with them. The divide between medicine and midwifery existed pre-COVID but is more exaggerated now and characterized by vitriol and intolerance. The midwives call their increased persecution by the Puerto Rican obstetric community “a witch hunt,” “a fear campaign,” and “a crusade against midwives.” The birth workers I spoke with agreed overall that more than anything, COVID has been making matters of reproductive health and justice more polarized. The virus is also making more evident the extreme structural inequalities between the wealthy and the poor that already existed but are more visible now, and more severe due to the fragile state of the economy during the pandemic.

As COVID has been making matters of inequality and injustice more visible—including the divide between midwives and the medical community and the economic disparities among clients--this visibility is integral to structural reform and change. For example, doula’s rights were temporarily restricted at the beginning of the pandemic, making many feel that the island’s maternity care system had “regressed,” “lost progress,” and “gone backwards” in a number of ways. They also worried that these changes would be difficult to reverse (and that the journey back would be just as long as the journey there had been). However, doulas, mothers, and allies alike rallied to have support persons recognized and protected, and they eventually were allowed to return back to the labor and delivery wards (as noted above)^10^. With disaster, there is inevitably destruction, but also a chance to rebuild. While there are still a number of barriers to overcome, midwives are glad that people are at least more aware of these options now and hope that families may embrace and fight for these alternatives in the future.

## Discussion: The Impacts of Disasters

In Puerto Rico, the impacts that COVID has had on birth have been very similar to the impacts of previous disasters—the hurricanes and earthquakes. During all of these events, midwives have been and continue to be primary disaster responders; home births have increased (either through choice or necessity—as is the case when people are physically unable to leave their homes); and pregnant people (and the general public) continue to become more aware of this alternative option. This growing awareness is due in large part to the Puerto Rican media coverage of midwifery care and home birth during disasters, which has continued to increase over these past three years (see section 2). Yet the sensationalization of infectious disease in the media has also instilled fear in soon-to-be, laboring, and new mothers that has prompted them to self-quarantine and made them hesitant to leave their homes. We are seeing this now with COVID, but we also saw this happen with Zika for a long time in Puerto Rico, especially around the time of Hurricane Maria when there were issues with flooding and standing water (National Institute of Health, 2017). These fears of infectious diseases (Zika, COVID) may not only prompt women to prefer home birth, they may actually lead to extreme isolation and restricted mobility outside of the home. The “turf war” between medical professionals and midwives, seen as well during the hurricanes and the earthquakes, persists and seems to intensify with each disaster as pregnant Puerto Rican women increasingly embrace the midwifery model of care.

So many of these impacts observed in Puerto Rico can also be witnessed in disaster responses worldwide. Globally, and historically, we see that midwives are often the first to respond in the immediate aftermath of disasters, as they are trained in “low-tech/skilled touch” (Davis-Floyd, 2021) methods and can make do with the most basic supplies. We have also seen destruction to hospitals and absence of medical professionals during these events, furthering the need for local birth workers’ assistance (ibid.). Among these events is our current COVID pandemic, which has had similar impacts and responses worldwide. In Puerto Rico, the US, and a number of nations, there has been an increase in demand for midwives, home births have become more popular and preferable as fear of COVID makes women wary of hospitals, and frustration over restrictions on support persons prompt women to abandon the medical model altogether (Davis-Floyd, 2021; Davis-Floyd et al., 2020). However, this rapid surge also causes a shortage of community birth workers, as many of the areas in which home births and midwifery are in high demand do not support and encourage midwifery nor facilitate home births. While the new-found appreciation for midwifery is certainly encouraging, serious changes need to be implemented if midwifery care and home births are to be truly accessible to all, and widely available during times of disaster, when their “low-tech, skilled touch” care is needed most. In order to facilitate this midwife disaster response, Wick and Hassan (2012) suggest “Planning for emergency care by mapping the location of midwives, supplying them with basic equipment and medications, and legitimizing their profession with an appropriate scope of practice, licensing, back-up, and incentives … ”

## Conclusion: Questions and Considerations

As I demonstrate in this article, COVID, among other disasters, has shifted a great deal of childbirth to the care of Puerto Rican community birth workers such as doulas and midwives. As the other articles in this special issue show, this is a pattern observed worldwide as well, and one that inspires hope for a future that embraces humanistic birth practices that incorporate necessary technologies (Davis-Floyd, 2018b; Davis-Floyd, 2021). However, this transition to home birth (in societies that do not facilitate it and are not designed for it) is not without its complications for both midwives and birthing women. In these uncertain times, there is still so much we are unsure of and so many questions that have yet to be answered. We will not know until we are truly on the other side of this pandemic, and have had the time to conduct more research, just how much it has impacted us and the women and children of our communities.

Moving forward, we will need to consider: How high did the home birth rate go during the pandemic? The induction and cesarean rates? What can be done about birth workers’ increased risk of contracting COVID? What are the impacts? Will the transition to home birth and midwifery be sustainable (with regard to government support, policy changes, and increased accessibility)? Will midwifery be altered by the pandemic, made more official and therefore more clinical? Will problematic hospital practices persist? And of course, the people of Puerto Rico are constantly asking “What about the next catastrophe?” As I write, they have been coping with more earthquakes, weathering severe storms, and are preparing for a hurricane season that they know will be complicated by this ongoing pandemic. It is already being hypothesized that COVID will make hurricane response more difficult, due to a declining economy, compromised infrastructure, and fear of spreading disease (Canales, 2020).

And, unfortunately, the people of Puerto Rico are not alone in asking the question, “What next?” Worldwide in this Anthropocene Era, we are seeing disasters, including epidemics and pandemics, increase as a result of human-driven climate change (Wallace-Wells, 2020; Davis-Floyd, 2021). Whether we are prepared or not, these events will continue to arrive, sometimes overlapping with one another. This is why it is so crucial that we study how disasters impact human health, including reproductive health. In understanding what impacts past disasters have had, and how they have been successfully managed (or mis-managed), we can better prepare for the future and hopefully ensure the health and wellbeing of mothers, babies, and birth workers everywhere.

## Data Availability

The datasets presented in this article are not readily available because this is qualitative (ethnographic) data rather than quantitative data. Research results take the forms of interviews/ quotes/ vignettes. Interviews and notes are still in raw form and have not been coded or analyzed. Requests to access the datasets should be directed to tuf60917@temple.edu

## Footnotes

1 See Davis-Floyd (2021) for descriptions of “low-tech, skilled touch” midwifery disaster care following the 2004 Aceh tsunami, the 2013 Hurricane Haiyan in the Philippines, and the Great Japanese Earthquake of 3/11/11.

2 It is worth noting that in recent years the home birth rate has risen to closer to 2% in the United States as a whole.

3 See Ivry et al. (2019) for a discussion of childbirth experiences *during* an earthquake (in Japan).

4 This preparation could potentially impact psychological and physiological birth preparedness.

5 Though, as previously discussed, during the pandemic some of my interlocutors are offering services for free or at reduced cost, and when responding to disasters such as the hurricanes and the earthquakes, they must volunteer and rely on donations.

6 Doulas (unlike independent midwives) can attend hospital births in Puerto Rico, do not serve as competition for “catching the baby,” and provide emotional and social support to patients that clinicians most often do not have the time for. In this way doulas may assist in their work without truly threatening it.

7 See Davis-Floyd and Johnson (2006) for a recounting of these same struggles in the mainland United States.

8 While doulas have been restricted during COVID from accessing clinical spaces, the emotional/social support that they provide in labor is still invaluable. Fearing having to choose between a partner or doula as the “one sole support person” (in the hospital) was a driving force behind the increase in preference for home birth—a space where doulas continue to be welcome.

9 See Hays and Prepas (2015) for a discussion of the institutionalization and standardization of midwives in disaster response.

10 See Yakovi Gan-Or (2020) for a discussion of the legislation surrounding birth assistants in the states (and globally) during the pandemic.
